# Conscious imagination vs. unconscious imagination: a contribution to the discussion with Amy Kind

**DOI:** 10.3389/fpsyg.2024.1310701

**Published:** 2024-07-25

**Authors:** Agnieszka Jaworska

**Affiliations:** Institute of Philosophy, Cardinal Stefan Wyszynski University in Warsaw, Warsaw, Poland

**Keywords:** imagination, predictive processing, conscious level, unconscious level, perception

In her paper entitled “*Can Imagination Be Unconscious?*”, Kind (2021) explores several approaches to the concept of unconscious imagination and claims that the thesis of unconscious imagination lacks strong argumentation. However, these perspectives consistently dichotomize consciousness and unconsciousness. She argues that the reasoning for unconscious imagination that have been put forward so far are not sufficiently strongly argued, and that the examples put forward by proponents of unconscious imagination are not sufficiently strongly argued. Furthermore, according to Kind, the situations they describe can be explained in a different way, using concepts already available in the literature, such as having beliefs or conscious imagining. I propose an alternative view, in which I will point out that Kind's argumentation is limited in scope. By employing the Predictive Processing framework, which posits the existence of unconscious imagination, I aim to assert that imagination can be considered unconscious under the assumption that the demarcation between consciousness and unconsciousness remains nebulous. Consequently, I will demonstrate that Kind's argument does not apply to such approaches to the imagination in which the boundary between conscious and unconscious states is blurred.

## Can imagination operate at the unconscious level?

Kind's paper delves into the intricacies of unconscious imagination, conceptualizing it as a specific, delineated activity that can commence and conclude. However, imagination can encompass a broader spectrum. It can be viewed as a cognitive faculty enabling a profound understanding of the world, especially regarding phenomena that are complex and not readily apparent (Gregory, 1970).

Unconscious imagination becomes apparent when our future experiences deviate from our mental projections. It is only when reality diverges from our imagined scenarios that we recognize the disparities (Blomkvist, 2022). The complexities of cognition and our engagement with the world necessitate vast knowledge, with certain aspects remaining beyond the reach of consciousness (Bowers et al., 1990).

In the contemporary landscape of cognitive sciences, the function of the imagination in cognition is defined in different ways. The approach proposed by, among others, Frascaroli (2021) advocates for the adoption of the Predictive Processing (PP) framework, as proposed by Hohwy (2013). According to PP, the brain constructs a virtual model of the world, known as the generative model, which generates predictions aimed at minimizing prediction errors—the disparities between the model and the actual world. This process enhances the effectiveness of an organism's actions in the world. This perspective allows us to conceive of imagination as an unconscious phenomenon, where all perceptions are intricately linked to imaginative processes occurring at the unconscious levels of the model.

The PP framework depicts the brain as an organ that produces a virtual reality that mimics the outside world. In this reality, sensory data is also virtual, with the task of anticipating stimuli from the world (Gładziejewski, 2016). When visually perceiving a particular object, what we get is sparse data—what we can see from a given angle and is sufficiently illuminated. When a Rubik's cube lies before us we see its three walls. However, the manufactured model of reality allows us to go beyond sensory data. We have ideas of what this cube looks like from the other side, what texture it has, how much it weighs.

Since imagination is required for perception in PP (cf.; Clark, 2015; Swanson, 2016) and since perceptual inference occurs unconsciously (cf. Helmholtz, 1948; Clark, 2016; Gładziejewski, 2016), we can say that our entire base of prediction-imagination is subjugated in a controlled hallucination (Perlovsky, 2007; Koenderink, 2010; Clark, 2016; Paolucci, 2021), with the goal of avoiding prediction error.

The process of perception from vague to clear is not accessible to our consciousness. As Perlovsky (2007) points out, only 0.01% of neuronal activity is conscious. As he goes on to point out, psychological experiments show that perceptions that appear fluid and conscious to us are largely based on “filling in the blanks”. I would argue that these fillings are imaginations. Imagination enters where conscious perception does not work. Since imaginations are representations without actual sensory stimulation, and conscious perception of a single object is supported by unconscious perceptual fills of the surrounding world, we can claim that perception is filled by representations created by imagination. As Perlovski argues, we can imagine a certain action with its consequences, it may be that these imaginings are fuzzy, vague, unclear and reach consciousness only after they “reach consciousness only after they converge to a “reasonable” course of action, which can be consciously evaluated” (Perlovsky, 2007, p. 129). Perlovski points out that it is impossible to consciously perceive all of reality, because the incoming stimuli are too many.

Arguments for the continuity of perception and imagination were demonstrated in their research by Kosslyn and Sussman (1994). Kosslyn's approach was developed by Grush, who enriched it with the Kalman filter. This understanding helps explain how the brain controls sensorimotor responses on an ongoing basis. The time it takes to send information from the brain to the muscles and then process that information is longer than the actual precise response (Grush, 2004; Francuz, 2007). The model of an organism's activity in the external environment produced by the brain allows predictions to be made based on previous predictions. Expanding Kosslyn's theory to include the equation of the Kalman function in terms of upward and downward processes allows us to identify two parts of the process—predictive and filtering. Grush's emulation theory also helps explain why the same system is responsible for both perception and imagery formation (Francuz, 2007). It is important to point out that Grush's concept refers to sensorimotor imagination, but I refer to it as an example of the application of Bayesian theories to describe the functioning and role of imagination.

Within the generative model, sensory stimuli elicit predictions organized hierarchically, ranging from low-level, such as changes in the visual field, to high-level predictions, like anticipating the sight of a dog. Our understanding of the world constantly evolves and adapts through Bayesian inference processes (Clark, 2013). The imaginative mechanisms in perception operate diversely (Kirchhoff, 2018). If a stimulus can be easily explained within the prediction hierarchy, imagination has a limited role. However, when interpreting the stimulus becomes intricate, perception shifts toward inference, resembling imaginative activity (Frascaroli, 2021). Imagination's involvement in cognition varies, forming a spectrum of imagination active in all cases, yet with varying intensity.

In her earlier works (Kind, 2020a,b), Kind posits that imagination is a skill honed through practice and can be consciously controlled. However, understanding the underlying cognitive architecture facilitating this process is crucial. Blomkvist (2022) advocates for incorporating Bayesian inference and Bayesian generation within the framework of PP. This framework highlights that imagination is not merely a skill, as proposed by Kind (2020a,b) (see also Anderson, 1982; Dreyfus, 2002; Fridland, 2014), but also a mechanism operating at the unconscious level through an approximation of the Bayesian selection mechanism. Blomkvist clarifies that the selection mechanism enables the extraction of pertinent representations from memory, which are then recombined and, through Bayesian generation, form imaginative constructs. Moreover, similar to predictions, imaginary representations can be adjusted when uncertainties arise concerning their correspondence with reality.

## Conclusion

In my view, Kind's analysis lacks consideration of models or frameworks like PP, which do not delineate a clear boundary between conscious and unconscious imagination. Imagination actively contributes to processes of knowledge production. Additionally, drawing a rigid distinction between having beliefs and imagining proves challenging. Imagination's role in cognition should be conceptualized as a spectrum, as it operates continuously, albeit to different extents, contingent upon the nature of the object under cognition. High-level conscious processes are inherently rooted in low-level unconscious ones, making it intricate to distinctly segregate conscious from unconscious processes.

It is essential to note that within the PP framework, the demarcation between conscious and unconscious states—that is, conscious and unconscious states—is ambiguous (Piekarski, 2017; see also Drayson, 2012). Simultaneously, asserting a clear boundary between perceptual and imaginal processes is challenging (Jones and Wilkinson, 2020). Consequently, distinguishing conscious imagination from its unconscious counterpart directly proves intricate. Hence, Kind's argument against unconscious imagination does not appear conclusively established. Accepting such a solution necessitates preliminary exploration within Kantian-inspired research frameworks in contemporary cognitive science, such as PP (cf. Swanson, 2016), or more broadly, through Bayesian modeling (cf. Brook, 2019; Gottwald and Braun, 2020).

## Author contributions

AJ: Writing – original draft, Writing – review & editing.
